# The Relationship between Urbanization, the Built Environment, and Physical Activity among Older Adults in Taiwan

**DOI:** 10.3390/ijerph15050836

**Published:** 2018-04-24

**Authors:** Nuan-Ching Huang, Shiann-Far Kung, Susan C. Hu

**Affiliations:** 1 Department of Urban Planning, College of Planning & Design, National Cheng Kung University, No.1, University Road, Tainan City 701, Taiwan; sunnynching@gmail.com (N.-C.H.); sfkung@mail.ncku.edu.tw (S.-F.K.); 2 Department of Public Health, College of Medicine, National Cheng Kung University, No.1, University Road, Tainan City 701, Taiwan

**Keywords:** urbanization, built environment, physical activity, older adults

## Abstract

Urbanization and ageing are global phenomena and offer unique challenges in different countries. A supportive environment plays a critical role in addressing the issue of behavioral change and health promotion among older adults. Many studies in the U.S., EU, and Australia have considered promoting physical activity in the community based on ecological models, whereas very few Asian studies have examined the relationships among urbanization, the built environment and physical activity in elderly at the ecological level, especially from a multi-level perspective. Due to the prevalence of post-war baby boomers and a very low birth-rate, the older population (aged 65 years old and older) in Taiwan has increased rapidly since 2011 and has exceeded the younger generation (0–14 years old) in 2017. Hence, the purpose of this study was first to examine the degree of urbanization in townships and the status of related built environments in Taiwan and then to investigate whether the built environment is associated with recommended amounts of physical activity among older adults. Three national datasets and a multi-level design were used in this research. Data at the individual level was obtained from the 2009 National Health Interview Survey (NHIS) which was taken from June 2009 to February 2010. Ecological data was obtained from the 2006 National Land Use Investigation of the National Geographic Information System and the 2010 Population and Housing Census. The analyses included a descriptive analysis, a bivariate analysis, a multiple logistic regression, and a multi-level analysis, utilizing a mostly hierarchical linear model (HLM). The results showed a significant relationship between factors at the environmental levels and physical activity in older adults. Urbanization, the built environment, and the median income of townships were positively correlated to the physical activity of the older adults. After controlling for individual-level factors, urbanization still exhibited this correlation. Parks and green spaces were associated with achieving the recommended amount of physical activity. However, there was no relationship after controlling for factors at the individual level. Detailed discussions were provided.

## 1. Introduction

Population ageing is an important public health issue and present unique challenges in many countries. By 2050, 66% of the world’s population will live in cities and 90% will reside in Asia and Africa. At least 22% of these populations will comprise older adults aged more than 65 [1,2]. Hence, cities have the responsibilities to balance their role as drivers of economic development and the quality of life of older residents.

Physical activity has been documented to reduce the risk of cardiovascular disease, hypertension, obesity, disability, stroke, type II diabetes, colon cancer and breast cancer, and all-cause mortality [3,4,5,6]. The health benefits of physical activity for older adults include improvements in muscle strength, psychological status, well-being and quality of life, cognitive function, social engagement, and social network [6,7,8,9]. According to a report from the World Health Organization, older adults should do at least 150 min of moderate-intensity physical activity or at least 75 min of vigorous-intensity physical activity or an equivalent combination of moderate- and vigorous-intensity activity throughout the week [10].

Factors associated with physical activity in previous studies have typically been focused at the individual level, such as demographics, psychological factors, health status, lifestyle, social support, and resource access [11,12,13]. However, many experts have suggested that health promotion needs to be conducted based on an ecological approach that considers individual and environmental levels [14,15,16,17]. Studies taking an ecological approach started in 2000 and addressed environmental factors only related to personal perceptions, such as crime [18,19] and traffic [18,19,20,21,22]. Recent environmental factors have included density/urbanization [12,23], greenery, scenery aesthetics [24,25], and parks and open space [25,26].

Noticeably, Barton and Grant proposed a health map that indicated the complex relationship among health, the environment (physical/social/economic), and the entire eco-system [27,28]. As the health map shows, the built environment is a key factor in ecological models in terms of influencing people’s behavior and wellbeing, which in turn is directly affected by the planning, design, construction and management of “spaces and channels” [29]. The term built environment in different disciplines has different definitions and interpretations. In health-related research, it comprises the buildings, spaces, and products that are created or modified by people [30,31,32]. Therefore, the built environment would be considered to be urban designs and element within them, land-use characteristics, recreational features, transportation systems (such as streets, foot and bike trails, public transit, and so on), and encompasses patterns of human activity within the physical environment [32].

In recent years, there has been increasing interest in understanding the association between the built environment and health-related behavior [33,34]. At least five research dimensions of the built environment were examined, including housing, transportation, food, parks and green space, and squalor [35,36]. Other factors related to the built environment such as density and intensity, land use mix, street connectivity, walkability, proximity, directness, and aesthetic qualities, have also been explored in related studies [32,35,37]. Measurement of the built environment can be grouped into 4 types: perceived (self-reported) [38,39], observational measures (systematic social observation, SSO, checklist or community audits) [40,41,42,43,44], government statistical reports [45,46], and geographic information system (GIS) measures [47,48,49].

Past research has indicated that the built environment is important to promote physical activity [50,51,52,53,54], but studies have shown equivocal results [33,55,56]. Fox examples, neighborhoods with greater amounts of greenspace were associated with weekly walking and moderate-to-vigorous physical activity (MVPA) in middle-to-older-aged adults. Participants living in neighborhoods with a large amount of green space have been found to engage in walking and MVPA more frequently [54]. A follow-up study with 15,632 individuals conducted in the UK indicated that older adults living in neighborhoods with more greenspace tend to decline less in terms of physical activity [49]. However, another 2011 investigation conducted in Japan provided mixed results for the relation between physical activity and the built environment. It was found that population density and the presence of parks or green spaces were positively related to the frequency of sports activities but had no relation to total walking time [56].

Urbanization is the process of the population moving from rural areas to urban areas, which leads to many changes in economic, social and environments [57]. The lifestyle and behavior of the population, as well as the demography of urban areas, will also change significantly along with industrial transformation. Studies have found that urbanization [58,59,60,61] and income [62,63,64] were related to levels of physical activities. However, few researchers have considered urbanization and built environment at the same time [64,65,66,67,68]. The use of a multi-level analysis to examine the effects of the built environment and physical activity has increased since 2005 [69]. While many studies in the U.S., the EU, and Australia have considered promoting physical activity for older adults based on an ecological model [33,34,49,54], very few studies in Asia have examined the correlation between the built environment and physical activity among older adults, especially from a multi-level ecological perspective [24,65].

Taiwan became an aging society in 1993, and will soon be classified as an aged society and super-aged society in 2018 and 2025, respectively. Due to the prevalence of post-war baby boomers and a very low birth-rate, the older population (aged 65 years old and older) in Taiwan has increased rapidly since 2011 and has now exceeded the younger generation (0–14 years old) in 2017 [70]. It is important to enable older adults to live independently and to help them avoid disabilities and experience enhanced quality of life during their retirement.

Nevertheless, residential density, urban fabric, land use patterns, and lifestyle in Asian countries are totally different from those in western countries. In Taiwan, land use patterns are more compact and mixed used by narrow pavement and by bicycles and scooters. Older residents usually engage in walking or participate in physical activities in parks and at schools. Studies have shown that urbanization is one of the significant factors that influence levels of physical activity among older adults but the results have been inconsistent [12,23,58,61], probably because of the different types and content of urbanization. Hence, the purpose of this study aims to examine the level of urbanization of townships and the related built environment status in Taiwan so as to investigate whether urbanization level and built environment are associated with the recommended amount of physical activity among the older adults.

## 2. Methods

### 2.1. Study Design and Dataset

In this study, three national datasets and a multi-level design were used to examine the relationship between the built environment and physical activity in older adults. The three national datasets included (1) the 2009 National Health Interview Survey (NHIS) in Taiwan, (2) the 2006 National Land Use Investigation of the National Geographic Information System, and (3) the 2010 population and housing census and statistics from the Ministry of the Interior.

**Figure 1:**
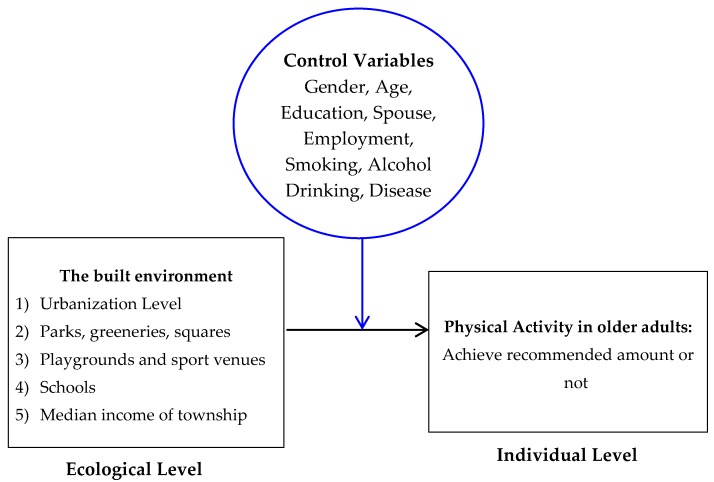
Study framework.

Data at the individual level was obtained from the 2009 National Health Interview Survey (NHIS) which was taken from June 2009 to February 2010. Ecological data was from the 2006 National Land Use Investigation conducted by the National Geographic Information System and 2010 Population and Housing Census. Both ecological datasets are taken every ten years so that the data from the last wave were taken in 1995 and 2000, respectively. Hence, this study used data for the 2010 population and housing census, which is the closest to the data from the 2009 National Health Interview Survey to analyze the level of urbanization. Figure 1 describes the study framework. A multi-level Hierarchical Linear Modelling (HLM) analysis was used in this study.

### 2.2. Measurement

#### 2.2.1. Individual Level

Individual data was from the 2009 Taiwan National Health Interview Survey (NHIS), which used a stratified multistage systematic sampling procedure and is a representative sample of each county and municipality in Taiwan. Samples were selected with probability proportional to population size. For this survey, a total of 25,636 participants (response rate of 83.96%) were interviewed covering topics including demographic information, health status, health services utilization, social participation, and behaviors such as smoking, drinking, and physical activity. The questionnaires were divided according to age group: under 12 years, 12–64 years, and over 65 years of age. A total of 2904 older adults aged 65 years and above was recruited in the survey.

##### Outcome Variable: Physical Activity

Amount of physical activity (minutes/week) was calculated using the following three questions:

(1) Did you exercise in the past two weeks? (0) No (1) Yes;
(2) How many times did you exercise for a minimum of 10 min in the past two weeks? Times;
(3) How long you did you spend each time you exercised? Hour Minutes.

According to the suggestion of World Health Organization, older adults should engage in moderate-intensity physical activity at least 150 min per week in order to improve health [10]. Because the distribution of PA was skewed (mean = 186.42, SD = 272.07, median = 60.0, skewness = 2.19, *p* < 0.001 by Kolmogorov-Smirnov test), this study used 150 min/week as the cut point to examine whether older adults have reached the recommended amount of physical activity (as regular PA).

##### Control Variables

Control variables at the individual level included gender, age (<70, 70–79, ≥80), educational level (illiterate, literacy, ≤6 years, ≥7 years), spouse (yes/no), employment (yes/no), smoking (yes/no), alcohol consumption (yes/no), no. of diseases (0, 1, ≥2).

#### 2.2.2. Ecological Level

##### Urbanization

Defining what is urban or constitutes an urban area is a difficult and fundamental issue. A majority of studies have typically used population size or density to classify regions as urban or rural because urbanization issues arise from demographics. However, urbanization includes not only changes in population size but also considers industrial transformation, land use pattern, government agencies, and facilities and services. The impacts of urbanization are multi-faceted, diverse, and complex both positively and negatively [71,72].

The main principles used to define the difference between an urban and rural environment or urbanization usually are based on population size, population density, location of governmental agencies, the percentage of economically active employed population, and specific features of urban environments such as government agencies, banks, and services [73,74,75,76]. However, demographic structure is not the motivational force leading to urban-rural change. Using only indicators related to demography is inappropriate especially in a rapid population structure transition period, such as the growth of aging population or a specific age group. Hence, four indicators were used in this study to analyze the urbanization level of townships including “no. of residents,” “the percentage of people working in secondary industries,” “the percentage of people working in tertiary industries,” and “population density.” A cluster analysis was used to examine the level of township urbanization.

##### The Built Environment

Three items of the built environment were examined in this research, including: (1) parks, greeneries, squares, (2) playgrounds and sport venues, and (3) schools, which are often used for physical activities [44,77]. Since the locations of parks and schools are usually in surrounding neighborhoods and free to access, walking is the most common pattern of physical activity among older adults in Taiwan.

All data was obtained from the 2006 National Land Use Investigation conducted by the National Geographic Information System. The national land use investigation in Taiwan was categorized into 9 types: agriculture, forest, transportation, water, public facilities, recreation, mineral and salt industries, and others. “Parks, greeneries, and squares” and “playgrounds and sport venues” are part of recreational land use, and schools are part of public facilities. Land use data was intersected and calculated using Arc GIS 10.3.1 (Esri Corporation, Redlands, CA, USA), and the unit of each township was calculated as per capita area (m^2^). In the analysis, the data was divided into quartiles because of the skewness (the lowest level of built environment served as the reference category) which has been used in other studies [49,78], and its relationship to the outcome variable was examined.

##### Control Variable

Only one external variable, median township income, was considered as an ecological level control variable. Data for median income of township was obtained from the 2006 tax statistics of the Ministry of Finance, which lists the comprehensive income tax of townships. In Taiwan, it is hard to obtain income data at the individual level. Hence, the median township income from the income declaration system was considered better than a survey. In this study, the median township income was divided into quartiles, where the unit for each township was calculated as per capita thousand dollars (NTD).

### 2.3. Participants and Procedures

**Figure 2:**
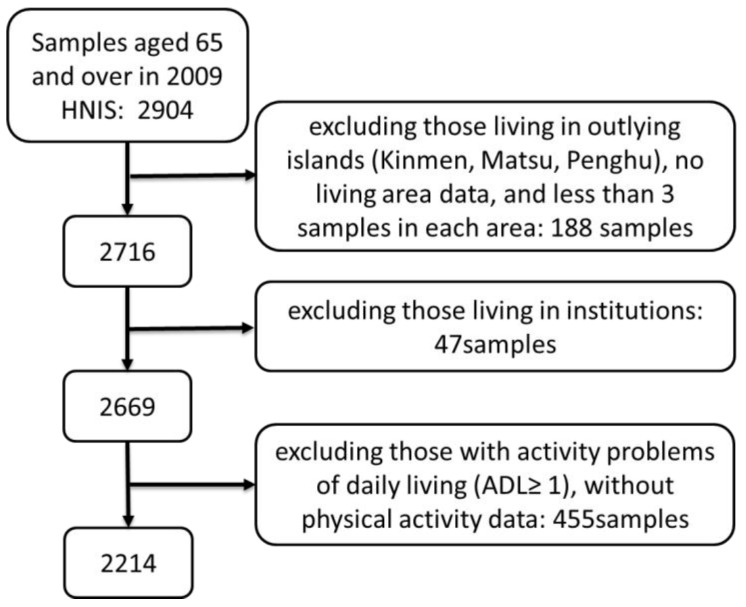
Participant enrollment procedure.

Figure 2 shows the sample enrollment procedure. In the beginning, a total of 2904 adults aged 65 and over in the 2009 NHIS survey was recruited as participants. After excluding those living in outlying islands (Kinmen, Matsu, and Penghu), no living area data, less than three samples in each area, institutional adults, activity problems of daily living (ADL ≥ 1), and without physical activity data, only 2214 adults aged 65 years and above were included in the analysis.

### 2.4. Statistical Analysis

The methodology of this study included three stages: firstly, we used census data to analyze the level of urbanization of townships in Taiwan with a cluster analysis method. Cluster analysis is an exploratory data mining task that groups homogeneity and heterogeneity variables. Variables with higher homogeneity (lower heterogeneity) were grouped. The standardized Z scores of each indicator were calculated for clustering. Ward’s minimum variance method and the squared Euclidean distance measurement were used in the cluster analysis.

In this study, the cluster analysis and related descriptive analysis were carried out with SPSS 17 software (SPSS Inc., Chicago, IL, USA). Secondly, the built environment data were obtained from the Land Use Investigation of Taiwan by using a GIS overlay analysis. Finally, ecological data (park and green spaces, playgrounds, sports venues, urbanization level, and income of townships) and individual data (2009 NHIS database) were merged to analyze the relationship between the built environment and the physical activity of the older adults.

The analyses in this research included a descriptive analysis, a bivariate analysis (Chi-square test and *T*-test), a multiple logistic regression, and a multi-level analysis, using a mostly hierarchical linear model (HLM). All analyses were carried out by using SAS 9.4 software (SAS Institute, Cary, NC, USA) and the PROC GLMMIX procedure [79].

Four models were built to examine the association of ecological and individual factors with regular PA by using multi-level analysis. Model 1 showed the influence of urbanization on regular PA; Model 2 examined the associations of urbanization and the built environment on regular PA; Model 3, based on model 2, adjusted for township income to understand effects of ecological factors on regular PA; and Model 4 was the full model which included all ecological and individual variables. A model for these estimation methods is described in the following equation, where *Y_ij_* is regular PA, *X_ij_* are individual *i*’s characteristics residing in *j* township, and *Z_j_* are ecological characteristics of *j* township:

(1) $$\mathrm{logit}\left\{P_{r}\left(Y_{ij}=1|X_{ij,}\;Z_{j}\right)\right\}=\gamma_{00}+\gamma_{10}X_{ij}+\gamma_{01}Z_{j}+\gamma_{11}X_{ij}Z_{j}+U_{1j}X_{ij}+U_{0j}+\varepsilon_{ij}$$

## 3. Results

### 3.1. Characteristics of Participants

Characteristics of the participants are listed in Table 1. The mean age of the participants was 74.74 ± 6.25 years. The majority of the 2214 participants was female, aged 70–79 years old, with no more than 6 years of education, with a spouse, unemployed, non-smoker, no alcohol consumption, engaging in physical activity and having more than one disease.

### 3.2. Urbanization Level and Built Environment

**Figure 3:**
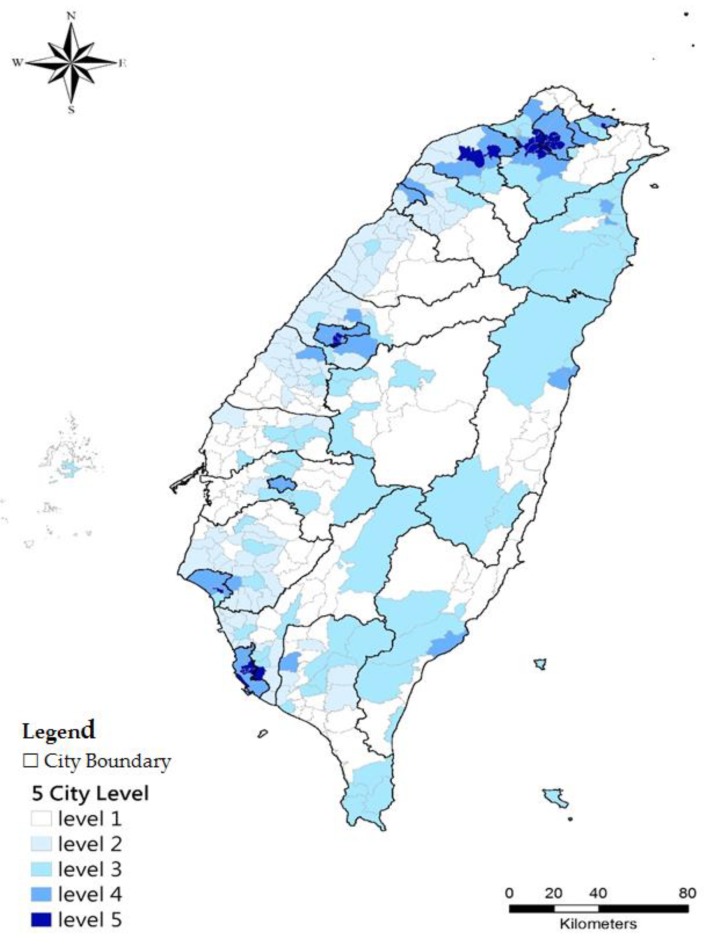
Distribution of urbanization levels of 358 townships.

Table 2 illustrates the urbanization level results. The township urbanization level was divided into 5 levels. Level 1 is the lowest urbanization level, which had the lowest population size (14,901) and density (329.6) but the highest primary industry percentage (17.68%). Level 5 is the highest level, which had the highest population size (213,150), population density (20,295.46), and the highest tertiary industry percentage (38.91%). The urbanization level distribution of 358 townships in Taiwan is mapped in Figure 3.

Table 3 reveals the descriptive statistics of the built environment for the 161 study townships from the 2009 NHIS. The distribution of the built environment in these townships is highly skewed. The median of the per capita area for “Parks and green spaces”, “Playgrounds and sports venues”, and “Schools“ at the township level are 2.92, 0.38, and 5.92, respectively.

### 3.3. Individual-Level Factors Associated with Regular PA

Table 4 shows the factors associated with achieving the recommended amount of physical activities per week for older adults. Nearly 40% of the older adults engaged in physical activity more than 150 min per week, and the percentage of males was significantly higher than females. Those who achieve the recommended amount of physical activity included being male, younger elderly individuals (less than 70 years old), more than 6 years of education, with a spouse, and unemployed. (Table 4).

### 3.4. Urbanization Level, Built Environments and Regular PA

The relationship between urbanization level, the built environment, and the recommended amount of regular PA in older adults is shown in Appendix A, Table A1. Opportunities to engage in the recommended amount of physical activity is shown to increase with urbanization level (OR = 1.31, 1.52, 1.91, 2.59 vs. 1.00, respectively). People who lived in urbanized areas from level 3 to level 5 were more than 1.5 times as likely to engage in the recommended amount of physical activity compared with those in level 1 (95% CI = 1.03–2.25, 1.32–2.77, 1.69–3.96, respectively). It provides a dose-response relationship between urbanization level and percent to reach the recommended amount of regular PA. People who lived in townships with park, greenery, and squares in Q3 group had nearly 1.5 times (OR = 1.46, 95% CI = 1.03–2.06) as much opportunity to reach the recommended amount of physical activity compared with those in the Q1 group. Opportunities to reach the recommended amount of physical activity was shown to increase with availability of playgrounds and sport venues, but the result was not significant. However, people who lived in townships with schools in the Q4 group had fewer opportunities to achieve the recommended amount of physical activity compared with those in the Q1 group (OR = 0.69, 95% CI = 0.48–0.98). Finally, people who had median incomes from townships in the Q3 and Q4 groups were more than 1.7 times as likely to achieve the recommended amount of physical activity compared with those in the Q1 group (95% CI = 1.27–2.46, 1.52–2.96, respectively).

### 3.5. Multi-Level Analysis

The results of the multi-level analysis at the ecological and individual levels are summarized in Table 5. Model 1 shows a dose-response relationship between urbanization level and PA, which shows that the higher level of urbanization has more chance to achieve regular PA. Model 2 provides the associations among urbanization level, the built environment, and regular PA. Older adults living in townships with parks, greenery, and squares in the Q3 group were 1.41 more likely to achieve regular PA, compared with those in the Q1 group (95% CI = 1.02–1.95). Those living in townships at urbanization levels 4 and 5 still had 1.76 and 2.8 times as much opportunities to achieve regular PA, compared with those in level 1 (95% CI = 1.17–2.65, 95% CI = 1.72–4.57), respectively. Model 3 examines the influence of ecological-level factors on regular PA, after adjusting for township income. Only urbanization level was a significant factor. Older adults living in the highest urbanization level were significantly positively associated with engaging in the recommended amount of physical activity compared with those in level 1 (OR = 2.14, 95% CI = 1.22–3.73). Those living in townships with parks, greenery, and squares in the Q3 group exhibited no relationship after controlling for median income in the township. Model 4 presents the full model containing all individual and ecological variables relating to the recommended amount of PA. Still, only the highest urbanization level remained a significant correlative factor, after controlling for variables at the individual level (OR = 1.90, 95% CI = 1.05–3.42). Noticeably, older male residents were found additionally to be more likely to engage in regular PA while compared with older females in the full model (OR = 1.31, 95% CI = 1.01–1.69). This presents the fact that ecological-level variables can modify the effects of individual-level variables.

## 4. Discussion

Consistent with previous ecological model research on the influence of physical activity, this study found a significant relationship between older adults’ physical activity and factors at the individual/demographic and environmental levels. This study had four only 3 listed important findings. First, five levels of urbanization in townships were demonstrated in Taiwan. Level 1 was the lowest urbanization level, and level 5 was the highest level. Second, factors associated with achieving the recommended amount of physical activity were found to be positively correlated with ecological variables, including urbanization level, park, greenery, squares, and median township income. Third, only the highest urbanization level was found to be positively associated with achieving the recommended amount of PA after controlling the individual variables.

### 4.1. Urbanization

Urbanization level tends to exhibit a hierarchical pattern. With increases in the degree of urbanization, the number of the built environment will decrease. Namely, the scope of services at higher levels of urbanization include the next lower level but also the vast rural areas, which shape the vertical division of the subordinate relationship [80].

These results are in accordance with recent studies indicating that older populations in urban areas are more likely to engage in physical activity than those in rural areas [58,81]. However, some studies indicated mix results for different levels of physical activity [23,61,82,83]. For example, the total amount of physical activity of older Icelanders was shown to not be associated with urban-rural areas. However, older Icelanders in urban areas has been found to be more likely to engage in leisure time physical activities. Thus, a significantly larger proportion of physical activity among rural older Icelanders is due to work as compared to older urban Icelanders [23]. Studies in China have also reported that older populations in urban areas tend to be more inactive than those in rural areas. However, the percentage of light physical activity was shown to be higher among rural residents as compared to urban dwellers. Namely, older adults living in rural areas reported less moderate and vigorous physical activity compared with those in urban areas [83].

### 4.2. Parks, Greeneries, Squares

**Figure 4:**
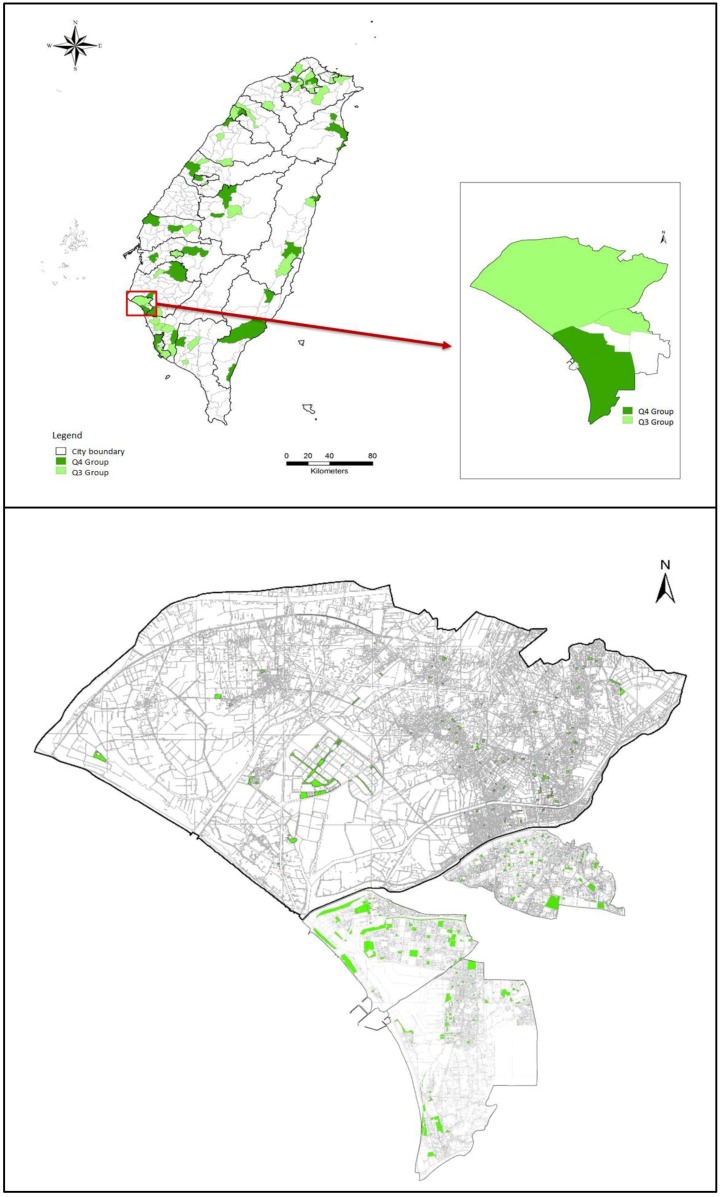
The distribution of parks and green spaces in the Q3 and Q4 groups.

“Parks, greenery, and squares” was significantly related to the achieved recommended amount of physical activity among older adults. Most interestingly, this positive relationship only appeared in the Q3 group and not in the Q1 group (OR = 1.46, 95% CI = 1.03–2.06). Therefore, we checked the GIS map to explore the differences for parks and green spaces between Q3 and Q4 groups and used Tainan City as an example (Figure 4).

Figure 4 indicates that parks and green spaces in the Q3 group were close to residential areas and were smaller. On the contrary, parks and green spaces in the Q4 group were larger and farther away. The price of land and the development of areas will affect the size of parks and green spaces. Normally, the size of parks and green space in high density areas is smaller than in suburban or rural areas. The location of big parks is usually far from residential areas, which makes it difficult for the older population to get to them. The barriers include things like requiring them to travel on roads with heavy traffic and uneven pavement, or other activities such as ball sports or bicycle riding in parks, poor light, difficulty finding toilets, and so on.

Several studies have indicated that utilization of parks is correlated with the size of parks and green spaces, the number of activities observed, the characteristics of users, the perceptions of park safety, and accessibility [77,84,85,86,87]. Cohen et al. [86] found that park size and the number of organized activities were associated with park use, but population density, poverty levels of neighborhoods, and perceptions of park safety were not. However, Kaczynski et al. [87] reported that parks with more features were positively associated with physical activity, but size and distance were not correlated. These findings were similar to those of Shores and West [84], who indicated that smaller parks had very low visitation rates, despite being located proximate to housing.

A study of Hong Kong elders reported that recreational walking was more prevalent than other forms of leisure-time physical activity [88]. Parks were associated with leisure-time walking, while gym/fitness centers were not associated with other forms of leisure-time physical activity. A qualitative study in Taiwan indicated that older adults mostly visit parks to participate in group exercises or walking and that the use of outdoor fitness equipment represents only a supplementary activity [89].

One possible explanation for the mixed findings on the relationship between park proximity and physical activity may be that park proximity might be associated with physical activity in different age groups or neighborhood environments. Furthermore, most research investigated park visitors but not those people who did not visit parks who lived in the surrounding parks and green spaces. Thus, it did not report the barriers related to those who did not visit these parks.

Facing a rapidly increasing aging population, it is important to understand the needs of the older population in the built environment, especially parks and green spaces. Future studies should review whether the features or characteristics of parks and green spaces meet the needs of older adults to improve the built environment as it relates to participation in physical activities in Taiwan.

### 4.3. Other Built Environments

Schools in the Q4 group showed a negative association with achieving the recommended amount of physical activity among older adults, but after controlling other ecological variables, there was no relation. Similarly, a study in Japan indicated that schools had no associations with the frequency of leisure time sports activity and total walking time [56]. Primary and secondary schools are very convenient spaces in Taiwan, so many people walk, play, and attend activities at schools. Due to the low birth rate, more and more primary schools have become abandoned, vacant, or unoccupied, especially in rural or remote areas. Therefore, activating such spaces and reusing schools is an alternate way to increase the amount of space available for activities suitable for older adults.

Older adults living in high income areas were found to be more likely to engage in physical activity. This finding was in line with previous studies. For instance, King’s research indicated that neighborhood income was significantly related with accelerometer-derived moderate to vigorous physical activity [63]. A study in Australia reported that old women living in high SES areas were more likely to do sufficient physical activity than those in low SES areas [90]. However, two U.S studies found that residents living in poverty areas were positively related with walking activity after controlling for individual SES [91,92]. These mixed results of area income may be due to the differences in the physical environment [64].

### 4.4. Individual-Level Factors

Factors at the individual level associated with physical activity in this study were similar to those of past studies. A higher proportion of older adults engaged in physical activity and met the recommendations if they had higher levels of education [93]. Older adults with one disease were found to be more likely to engage in physical activity than was the case for those with no disease or more than 2 diseases [94]. However, this finding was in contrast to an investigation in China [95]. In addition, older adults aged more than 80 years [7] who were employed [44] were more likely to be inactive. Gender differences matched those observed in earlier studies. This study found that 42.9% of the males attained the recommendations for practicing physical activities, as compared to only 36.9% of the females. This is consistent with Shibata’s research [96]. It is also in agreement with Salvador’s findings, which showed that older males are more likely to meet the recommended guidelines after controlling for ecological variables [97].

## 5. Contributions and Limitations

This study is the first interdisciplinary analysis of a national data connection between the National Land Use Investigation and the National Health Interview Survey in Taiwan and also the first to explore the relationship between the built environment and physical activity for older adults. Consequently, this data set provided sufficient samples and better quality of information by which to examine the correlation of interest.

Some important strengths were present in this study. To our knowledge, this was the first study using a multi-level analysis to examine physical activity among older adults from an ecological perspective for future intervention in Taiwan. This research provides empirical evidence of a relationship between the built environment and physical activity in Asia and also considers urbanization and the built environment at the same time. Land use pattern and healthy behavior of older adults are different from those in western countries. Based on this result, city planners could consider how to plan a suitable built environment for older adults and even consider how to reuse spaces in neighborhoods to make them more suitable for older adults.

This was a cross-sectional study, so some inherent limitations must be considered, including difficulty explaining the causality of related factors. The years covered by the three national datasets in the study are inconsistent. For example, the survey of population, housing census, and statistics was conducted in 2010. National Land Use Investigation was investigated in 2006, which is 3 years earlier than the 2009 National Health Interview Survey. Thus, the differences between survey years may have some potential effects to the full model. However, an effort was made in this study to compare the data years as close as possible.

Objective measures of the built environment were collected by using GIS, but the scale is not the smallest scale due to the fact that the living areas at individual data could only be at the township level. A smaller scale of living areas should be established to examine the relationship more precisely. Also, a buffer analysis in GIS could be used to obtain more precise information, such as friendliness of roads and sidewalks. Finally, a lack of related information in the township level, such air pollution, transportation, street walkability, crime, social cohesion and supportive groups in the living areas, is the potential limitation of this study.

## 6. Conclusions

Urbanization and the median income of townships were positively correlated to the physical activity of the older adults. Urbanization still exhibited this correlation after controlling for the individual level. Parks and green spaces were associated with achieving the recommended amount of physical activity. However, there was no relationship after controlling for factors at the individual level. In future research, we suggest clarifying the features and characteristics of parks and green spaces so as to compare the differences between subjective and objective environments as they relate to participation in physical activities on the part of older adults. In addition, comparative investigations of objective and subjective environments should be conducted in the future to provide planners with the ability to review the features of parks and green spaces as they relate to the needs of the older population.

According to the study results, the highest urbanization area, size, and locations of built environment are the important factors need to be considered in ecological level. It is suggested that governments, designers, and planners need to compare and clarify the advantages and barriers of the built environment features in different urbanization level for physical activity participation among the older adults. Furthermore, the gender difference between the built environment features and physical activity pattern and the barriers related to those who did not visit the built environment need to be taken into account. In addition, we suggest National Health Interview Survey should provide at least a small scale of living area or more accurate locations to connect more information relating to other environmental factors and the friendliness of the built infrastructure.

## Figures and Tables

**Table 1:** Characteristics of participants (*n* = 2214).

Variables	*n*	%
Gender		
Male	1003	45.3
Female	1211	54.7
Age (years)		
<70	770	34.8
70–79	1070	48.3
80+	374	16.9
Level of education		
Illiterate	616	28.0
Literate	375	17.1
≤6 years	669	30.4
≥7 years	540	24.5
Spouse		
Yes	1461	66.0
No	753	34.0
Employment		
Yes	390	17.6
No	1822	82.4
Smoking		
Yes	532	24.0
No	1682	76.0
Alcohol consumption		
Yes	859	38.8
No	1355	61.2
No. of diseases		
0	843	38.1
1	853	38.5
≥2	518	23.4
Amount of PA (min/week)		
0 (no PA)	1101	45.6
<90	156	7.1
90–149	169	7.6
≥150	878	39.7

**Table 2:** Urbanization level of 358 townships using cluster analysis.

Urbanization Level	No. of Township	Total Population	Population Density	Types of Industry (%) *		
				Primary Industry	Secondary Industry	Tertiary Industry
Level 1: lowest	118	14,991	329.3	17.7	11.8	22.0
Level 2	93	39,842	1042.6	5.9	28.3	22.4
Level 3	70	43,327	820.3	6.3	16.3	33.9
Level 4	49	167,300	5183.9	0.8	19.8	35.9
Level 5: highest	28	213,150	20,295.5	0.2	14.1	38.9

* Primary industry: including agriculture, forestry, fishing, and animal husbandry. Secondary industry: including manufacturing, building construction, engineering, and mining. Tertiary industry: including a wide range of service industry or businesses, such as financial and insurance industries, wholesale and retail, transportation and communications, intermediary, and catering services.

**Table 3:** Descriptive statistics of the built environment in the study townships (*n* = 161).

Built Environments	Mean	SD	Min.	Max.	Q1	Median	Q3
Parks, greenery, and squares	4.02	4.42	0.16	30.26	1.56	2.92	4.63
Playgrounds and sports venues	0.71	1.12	0.00	11.85	0.22	0.38	0.79
Schools	7.45	6.52	1.27	54.76	3.92	5.92	8.60

Unit: Per capital area (m^2^).

**Table 4:** Factors associated with the recommended amount of PA among older adults in Taiwan.

Variables	Achieve		Not Achieve		χ^2^
	*n*	%	*n*	%	
Gender					
Male	431	49.1	572	42.8	8.42 **
Female	447	50.9	764	57.2	
Age (years)					
<70	336	38.2	434	32.5	13.48 ***
70–79	421	48.0	649	48.6	
80+	121	13.8	253	18.9	
Level of education					
Illiterate	188	21.4	428	32.4	79.47 ***
literate	125	14.3	250	18.9	
≤6 years	268	30.6	401	30.3	
≥7 years	296	33.8	244	18.4	
Spouse					
Yes	623	71.0	838	62.7	16.00 ***
No	255	29.0	498	37.3	
Employment					
Yes	112	12.8	278	20.8	23.82 ***
No	766	87.2	1056	79.2	
Smoking					
Yes	208	23.7	324	24.3	0.09
No	670	76.3	1012	75.7	
Alcohol consumption					
Yes	345	39.3	514	38.5	0.15
No	533	60.7	822	61.5	
No. of diseases					
0	317	36.1	526	39.4	5.70
1	365	41.6	488	36.5	
≥2	196	22.3	322	24.1	

Achieve: physical activity ≥150 min/week. Not achieve: physical activity <150 min/week; ** *p* < 0.01, *** *p* < 0.001.

**Table 5:** Factors associated with the recommended amount of PA*: multi-level analysis.

Variables	Model 1			Model 2			Model 3			Model 4		
	OR	95% CI		OR	95% CI		OR	95% CI		OR	95% CI	
Urbanization												
Level 1: lowest	1			1			1			1		
Level 2	1.31	0.87	1.99	1.29	0.84	1.99	1.20	0.77	1.85	1.12	0.71	1.77
Level 3	**1.52**	**1.03**	**2.25**	1.45	0.97	2.16	1.35	0.90	2.02	1.26	0.82	1.92
Level 4	**1.91**	**1.32**	**2.77**	**1.76**	**1.17**	**2.65**	1.40	0.88	2.23	1.23	0.75	2.01
Level 5: highest	**2.59**	**1.69**	**3.96**	**2.80**	**1.72**	**4.57**	**2.14**	**1.22**	**3.73**	**1.90**	**1.05**	**3.42**
Parks, greenery and squares												
Q1: lowest				1			1			1		
Q2				1.08	0.77	1.50	1.02	0.73	1.43	1.00	0.70	1.42
Q3				**1.41**	**1.02**	**1.95**	1.31	0.94	1.83	1.29	0.91	1.83
Q4: highest				0.99	0.71	1.39	0.95	0.68	1.33	0.94	0.66	1.34
Playgrounds & sports venues												
Q1: lowest				1			1			1		
Q2				1.02	0.72	1.44	0.99	0.70	1.40	1.03	0.72	1.48
Q3				1.15	0.81	1.64	1.13	0.80	1.60	1.17	0.81	1.69
Q4: highest				1.24	0.87	1.77	1.18	0.83	1.69	1.28	0.88	1.87
Schools												
Q1: lowest				1			1			1		
Q2				1.14	0.79	1.64	1.15	0.80	1.66	1.21	0.83	1.78
Q3				1.14	0.78	1.68	1.15	0.78	1.67	1.22	0.82	1.81
Q4: highest				1.06	0.71	1.59	1.09	0.73	1.63	1.12	0.74	1.71
Income of Townships												
Q1: lowest							1			1		
Q2							1.11	0.80	1.55	0.97	0.68	1.37
Q3							1.36	0.92	2.00	1.20	0.80	1.80
Q4: highest							1.46	0.96	2.24	1.25	0.80	1.96
Gender (ref: Female)												
Male										**1.31**	**1.01**	**1.69**
Age (ref: <70 years old)												
70–79										0.85	0.69	1.06
80+										**0.57**	**0.43**	**0.77**
Education (ref: Illiterate)												
Literate										1.04	0.77	1.41
≤6 years										**1.38**	**1.06**	**1.80**
≥7 years										**2.21**	**1.64**	**2.97**
Spouse (ref: No)												
Yes										**1.33**	**1.07**	**1.63**
Employment (ref: No)												
Yes										**0.50**	**0.38**	**0.66**
Smoking (ref: No)												
Yes										0.84	0.64	1.09
Alcohol drinking (ref: No)												
Yes										0.85	0.68	1.06
No. of diseases (ref: 0)												
1										**1.41**	**1.14**	**1.75**
≥2										0.97	0.75	1.24

* Recommended amount of PA: ≥ 150 min/week. The bold number means statistically significant.

## Appendix A

**Table A1:** The association of each ecological factors with regular PA.

Categories	Urbanization Level				Parks, Greenery, and Squares *				Playgrounds and Sports Venues *				Schools *				Median Income of Township ^#^			
	*n*	OR	95% CI		N	OR	95% CI		*n*	OR	95% CI		N	OR	95% CI		*n*	OR	95% CI	
Level 1: lowest	23	1																		
Level 2	31	1.31	0.87	1.99																
Level 3	35	**1.52**	**1.03**	**2.25**																
Level 4	45	**1.91**	**1.32**	**2.77**																
Level 5: highest	27	**2.59**	**1.69**	**3.96**																
Built environment																				
Q1: lowest					41	1			40	1			40	1			38	1		
Q2					39	1.13	0.80	1.61	41	1.08	0.75	1.54	41	0.92	0.64	1.33	42	1.29	0.92	1.8
Q3					41	**1.46**	**1.03**	**2.06**	39	1.18	0.82	1.70	39	0.75	0.52	1.07	39	**1.77**	**1.27**	**2.46**
Q4: highest					40	0.98	0.69	1.39	41	1.22	0.85	1.74	41	**0.69**	**0.48**	**0.98**	42	**2.12**	**1.52**	**2.96**

* Per capital area (m^2^); ^#^ Per capita thousand dollars. The bold number means statistically significant.
